# Neural encoding and production of functional morphemes in the posterior temporal lobe

**DOI:** 10.1038/s41467-018-04235-3

**Published:** 2018-05-14

**Authors:** Daniel K. Lee, Evelina Fedorenko, Mirela V. Simon, William T. Curry, Brian V. Nahed, Dan P. Cahill, Ziv M. Williams

**Affiliations:** 1 Department of Neurosurgery, Massachusetts General Hospital, Harvard Medical School, Boston, 02114 MA USA; 2 Department of Psychiatry, Massachusetts General Hospital, Harvard Medical School, Boston, 02114 MA USA; 3 Harvard Program in Speech and Hearing Bioscience, Cambridge, 02138 MA USA; 4 Massachusetts Institute of Technology, McGovern Institute for Brain Research, Cambridge, 02139 MA USA; 5 Department of Neurology, Massachusetts General Hospital, Harvard Medical School, Boston, 02114 MA USA; 6 Harvard-MIT Division of Health Sciences and Technology, Boston, 02115 MA USA; 7 Program in Neuroscience, Harvard Medical School, Boston, 02115 MA USA

## Abstract

Morphemes are the smallest meaning-carrying units in human language, and are among the most basic building blocks through which humans express specific ideas and concepts. By using time-resolved cortical stimulations, neural recordings, and focal lesion evaluations, we show that inhibition of a small cortical area within the left dominant posterior–superior temporal lobe selectively impairs the ability to produce appropriate functional morphemes but does not distinctly affect semantic and lexical retrieval, comprehension, or articulation. Additionally, neural recordings within this area reveal the localized encoding of morphological properties and their planned production prior to speech onset. Finally, small lesions localized to the gray matter in this area result in a selective functional morpheme-production deficit. Collectively, these findings reveal a detailed division of linguistic labor within the posterior–superior temporal lobe and suggest that functional morpheme processing constitutes an operationally discrete step in the series of computations essential to language production.

## Abstract (web summary)

Functional morphemes allow us to express details about objects, events, and their relationships. Here, authors show that inhibiting a small cortical area within left posterior superior temporal lobe selectively impairs the ability to produce functional morphemes but does not impair other linguistic abilities.

## Introduction

Morphemes are the smallest units in human language capable of conveying particular meaning. For example, the word “talked” not only describes a conversation event, but also importantly places this event in the past. The selection of an appropriate inflectional morpheme, such as “ed” in “we talked” or “s” in “he talks”, is therefore critical to our ability to precisely convey the intended meanings. This process occurs rapidly in natural speech and comprises a core step in a series of linguistic computations that begin with formulating a thought and end with articulating the utterance^1–6^. Examining this process could provide important insights into the neural processes through which humans express specific word meanings and the relationships among words.

Prior neuroimaging studies have demonstrated that many brain regions of the left-lateralized fronto-temporal language network, including both the inferior frontal and posterior temporal/parietal regions, are active during word and sentence production^7,8^. Large portions of this language network plausibly store our linguistic knowledge representations (i.e., the mappings between meanings and linguistic forms), which are likely spatially distributed^9,10^ and accessed during both comprehension and production^11^.

In spite of these shared-knowledge representations, however, an important asymmetry exists between comprehension and production. The goal of comprehension is to infer the intended meaning from the linguistic signal. Abundant evidence now suggests that the interpretation is affected by both bottom-up, stimulus-related information and top-down expectations, and the representations we extract and maintain during comprehension are probabilistic and noisy^12–17^. In contrast, in production, the goal is to express a particular meaning, about which we generally have little or no uncertainty. To do so, we have to utter a precise sequence of words where each word takes a particular morpho-syntactic form, and the words appear in a particular order. These pressures for precision and for linearization of words, morphemes, and sounds might lead to a clearer temporal and/or spatial segregation among the different stages of the production process, and, correspondingly, to functional dissociations among the many brain regions that have been implicated in production^7,8^, compared to comprehension, where the very same brain regions appear to support different aspects of the interpretation (like understanding individual word meanings and inferring the syntactic/semantic dependency structure)^18–20^.

Although a number of sophisticated cognitive models of language production that specify the different stages and the relationships among them have been proposed^1–5,21^, understanding the precise neural mechanisms by which humans encode and time-causally enact different aspects of a linguistic message––including the division of labor spatially (within and across brain regions) and temporally (across time)––has proven to be a major challenge. Functional brain-imaging methods generally do not possess the temporal resolution needed to evaluate the individual components involved in word generation. They also do not afford causal inferences and, although lesions from natural trauma or strokes have critically informed models of language production^5,22^, they commonly affect extensive cortical areas as well as their underlying white matter tracts, complicating interpretation. Further, to the extent that the same brain region supports different stages of language production, a permanent lesion to a region would not allow for temporal differentiation of those stages. Against the backdrop of the remarkable progress in our understanding of language, the precise contributions of distinct cortical areas to different aspects of word and sentence production, such as functional morpheme processing, remain poorly defined.

Here, we examined a core language hub––the posterior superior temporal gyrus (P-STG)––in the encoding and production of functional morphemes (e.g., selection of “ed” in the word “talked”). Prior work has linked production processes to the so-called dorsal stream^23–25^, which connects regions in the posterior superior and middle temporal cortices with the frontal regions that support speech articulation^26–29^. Posterior temporal regions thus could (i) serve as the site where morpho-syntactic encoding takes place, and (ii) provide input to the articulation system in the frontal cortex. Indeed, although historically “Wernicke’s area”, which broadly encompasses the P-STG, as well as angular and supramarginal gyri (although this term has been used too loosely in the literature over the years^30^), has been linked with lexico-semantic processing and comprehension deficits^31–34^, some recent work has questioned its role in comprehension^35^, and a number of studies over the years have reported syntactic production deficits in patients with lesions that overlap with P-STG^36–40^.

In this work, we combined time-resolved cortical inhibition, acute neural recordings, and focal gray matter lesion evaluation to examine the potential role of P-STG in functional morpheme production. We find a focal cortical area below the left TPJ, whose inhibition transiently disrupts morphological morpheme selection, without distinctly affecting semantic and lexical retrieval, articulation, or basic language comprehension. Furthermore, we demonstrate that this region encodes syntactic error and word-form morphology, but not lower-level word properties or sentence-level lexico-semantic incompatibilities. Finally, we identify focal gray matter lesions in the P-STG that were associated with a selective functional morpheme-production deficit.

## Results

### Word-production task

Before discussing the findings, we briefly outline our approach. To obtain a detailed evaluation of cortical function and selectivity, we introduced a structured word-production task in neurosurgical participants undergoing planned intraoperative neurophysiology. A total of 27 participants were included (*n* = 14 for stimulation, *n* = 5 for recording, and *n* = 8 for lesion evaluation; Supplementary Table 1), and all were confirmed to have intact language function by preoperative performance (97.5% performance across task conditions; see further below, paragraphs 2–6). All parts of the study were performed in strict adherence with our institutional guidelines and detailed care was taken to ensure that they did not perturb any aspect of ongoing clinical care.

**Fig. 1:**
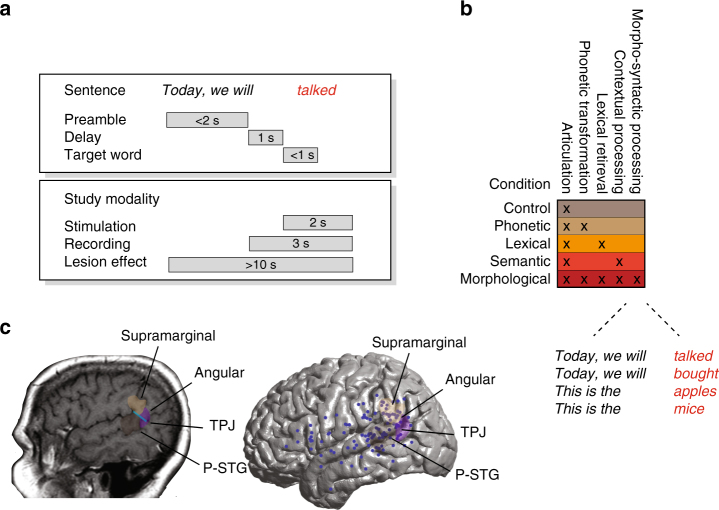
Task design and distribution of stimulation sites. **a** On each trial, participants were presented (auditorily) with a preamble such as “Today, we will ...”. After a 1-s delay, they were given a target word, presented visually (e.g., “talked”) that they had to produce out loud either as presented or after manipulating it in some way. **b** Several target word manipulations were introduced in order to test for the specific effect of stimulation on basic sensory perceptual processing and articulation (control condition), the ability to recognize and phonetically transform the target word (phonetic condition), the ability to retrieve lexical information from memory (lexical condition), and, more broadly, the ability to understand language and respond with the appropriate semantic knowledge (semantic condition). Additional comparisons included nouns vs. verbs, regular vs. irregular inflections, and suffix additions vs. subtractions (Supplementary Table 2). A few representative examples of sentence preambles (black italic) and target words (red italic) in the critical syntactic condition are provided. **c** Here, we focused on the posterior superior temporal cortex, which borders the supramarginal and angular gyri (P-STG refers to the posterior-most aspect of the superior temporal gyrus). The anatomical temporal–parietal junction (TPJ) is drawn in blue. Individual, stereotactically identified stimulation sites are shown by dark-blue dots (*n* = 113) on the right. Due to surgical constraints, however, not all stimulated sites were comprehensively tested (*n* = 54, “Methods”)

On each trial, participants were presented (auditorily) with a sentence preamble, followed by the visual presentation of a target (to-be-spoken) word, which had to be variably manipulated prior to articulation (Fig. 1a). To evaluate for processes selectively involved in the production of functional morphemes, five manipulations were tested (Fig. 1b). In the control condition, the target word was presented in the correct form and participants had to simply articulate it out loud. For example, given the sentence “Yesterday, we [talked_target_]”, they would have to say “talked”. By comparison, in the syntactic condition, the target word was presented in a form that was structurally incompatible with the sentence context, and participants had to provide the appropriate morphological word form to make it compatible with the sentence preamble. For example, given the sentence “Yesterday, we [talk]”, participants would have to say “talked”. Therefore, in both the syntactic and control conditions, participants had to understand the sentence preamble, as well as recognize and articulate the target word. However, the syntactic condition additionally required participants to morphologically manipulate the target word prior to articulation in order to make the utterance well-formed.

To examine the potential effects of task demands and test whether difficulty may be restricted to cases where a morpheme had to be added^41^, we included both suffix additions (e.g., change “talk” to “talked”) and subtractions (e.g., change “talked” to “talk”) (Supplementary Table 2). We also compared the participants’ preoperative performance to their performance during stimulation testing in order to evaluate whether small between-condition differences in difficulty may have potentially influenced stimulation results (i.e., even though performance was near-ceiling preoperatively, certain sentences elicited more errors; Supplementary Figure 1). Further, for generalizability, noun and verb targets were given in equal proportion (mismatching in tense for verbs or in number for nouns). We also included both regular forms where the correct inflection follows a rule (e.g., adding "-ed" to form the past tense) and irregular forms, where the correct word-form plausibly has to be retrieved from memory^42^.

Next, to examine the participants’ ability to perform any word-form transformation or simply identify any incompatibility or “error” in the target word, we included a phonetic condition. Participants were given the target word with the suffix marked in bold and were instructed to omit the bolded morpheme irrespective of whether the resulting word form would be appropriate in the context. For example, given the sentence “Yesterday, we [talk**ed**]”, they would have to say “talk” (cf. a similar trial in the syntactic condition: “Today, we will [talked]”, to which they would also have to say “talk”).

Lastly, to examine the participants’ ability to access any lexical information, we included trials where they had to name animate and inanimate objects. For instance, they may be given the preamble “This is a” followed by a picture of a table (lexical condition). In addition, and more broadly, to examine the participants’ comprehension and their ability to flexibly access appropriate semantic knowledge based on the preamble context, we introduced a semantic condition. Here, for example, given the preamble “Today, the month is”, they would have to provide the appropriate month of the year.

In summary, our tasks tapped a variety of mental processes engaged during word production, from semantic–conceptual retrieval, to accessing particular words, to inflecting those words or selecting the appropriate stored word form, to, finally, articulation. This design, therefore, allowed us to begin to examine intraoperatively the time-causal contribution and selectivity of neural processes that support functional morpheme production.

### Functional selectivity revealed by focal cortical inhibition

The surface contact delivery of focal bipolar currents allows for a brief, localized, and reversible inactivation of small cortical areas on the spatial order of a cubic centimeter in individuals with otherwise intact language function (see “Methods” for additional details). Here, stimulation was given for 2 s and was time locked to the presentation of the target word. Speech production was recorded and time-aligned with a microphone. Fourteen participants underwent stimulation in a total of 54 registered sites (Fig. 1c; “Methods”), and deficits for each site were defined based on whether the correct target word was produced in the control, syntactic, phonetic, lexical, and/or semantic conditions.

**Fig. 2:**
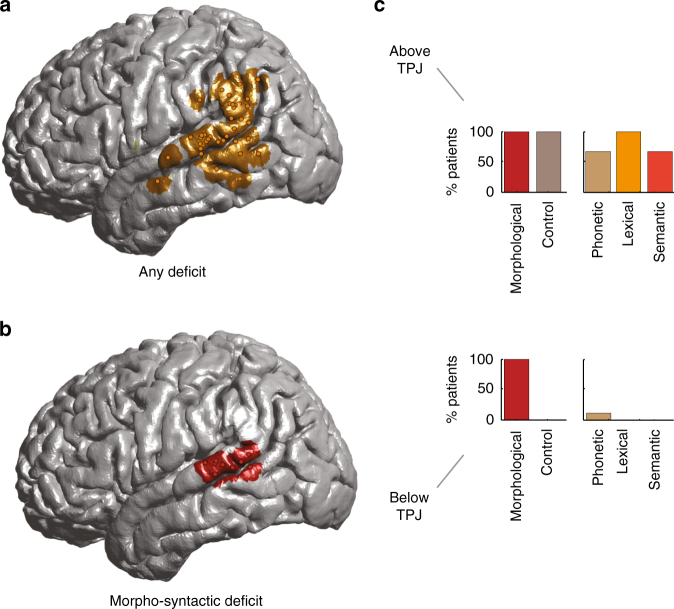
Stimulation of focal sites transiently inhibits functional morpheme production. Individual stimulation sites are defined based on their selectivity and anatomic location. Here, the stimulation sites were evaluated across all the participants. **a** Stimulation sites that produced a non-selective linguistic deficit during production. **b** Stimulation sites that produced a deficit during the syntactic condition only. **c** The bar graphs represent the percentage of patients stimulated in the stated region in which stimulation elicited a deficit (out of those that produced any significant deficit) above and below the TPJ (see Fig. 3, below, for further details and Supplementary Figure 2)

We first focused on the syntactic condition. Of the 14 participants in which stimulation was given, nine had craniotomies that provided direct access to the P-STG. Of these, we identified a single site in each of nine participants (*n* = 9 sites) in which stimulation elicited significantly more errors in the syntactic condition compared to the control condition (binomial test, *n* ≥ 5, *p* < 0.0001, compared to the preoperative baseline, Bonferroni corrected for testing across multiple sites; Fig. 2a, b). Overall, stimulation of these sites led to an incorrect response on 55.6% of trials in the syntactic condition but only on 6.4% of trials in the control condition (chi-square test, *n* = 117, *χ*^2^ = 26.6, *p* < 5 × 10^−7^). For example, stimulation of these sites produced an incorrect response when the participants were required to produce the word “talked” after being given the preamble “Yesterday, we [talk]”.

Stimulation in these sites led to a selective deficit in functional morpheme production. First, performance during the phonetic condition, in which the morpheme had to be simply manipulated based on an instructed cue, was not different from the control condition (11.8% vs. 6.4%; chi-square, *n* = 79, *χ*^2^ = 0.50, *p* = 0.48; Fig. 2c). Moreover, this result was not due to differences in task difficulty or demand as largely identical results were obtained for sentences that elicited a higher (2–4%) vs. lower (0–2%) error rate preoperatively at baseline (*t*-test, *n* = 138, *t* = –0.52, and *p* = 0.60). We also observed no difference in performance during stimulation based on whether the suffix had to be added or removed (“talk” → “talked” vs. “talked” → “talk”; chi-square, *n* = 138, *χ*^2^ = 0.26, *p* = 0.69) or whether the target word was a noun or a verb (chi-square, *n* = 138, *χ*^2^ = 0.1, *p* = 0.93; see additional controls in paragraph 4).

Second, we tested whether stimulation of these sites may have produced a broader disruption in lexical processing. However, we found no difference in performance in the lexical compared to control condition (0% vs. 6.4%; chi-square, *n* = 78, *χ*^2^ = 1.7, *p* = 0.18; Fig. 2c and Supplementary Figure 4). In addition, while regular forms can be assembled on-the-fly by following simple morpho-syntactic rules (e.g., append “-ed” to put the verb in the past tense), irregular forms plausibly have to be stored as separate lexical entries^42^ and should thus place greater demands on lexical retrieval mechanisms. Yet, we found no difference in the syntactic condition between regular and irregular target words (chi-square, *n* = 138, *χ*^2^ = 1.1, *p* = 0.29; see also ref. ^43^, which shows that meta-analysis of human subjects with Broca’s aphasia failed to display a consistent pattern of irregular and regular inflectional morphology).

Finally, we examined whether stimulation could have affected more general cognitive processing and semantic retrieval; for example, the ability to understand the contextual relation between the preamble and the appropriate word to be spoken. To this end, we examined the semantic condition trials, where participants had to retrieve semantic facts from memory, but similarly found no difference when compared to the control condition (8.5% vs. 6.4%; chi-square, *n* = 81, *χ*^2^ = 1.06, *p* = 0.32; Fig. 2c and Supplementary Figure 4).

Therefore, stimulation of these sites appeared to disrupt the participants’ ability to apply the appropriate morpheme or retrieve the correct word form in order to convey the appropriate meaning given the context. However, stimulation did not disrupt their ability to recognize, process, or articulate the words. It also did not appear to affect their ability to simply phonetically manipulate the word suffix, or their ability to retrieve from memory lexical and semantic content. We therefore conclude that these sites were essential for selecting the appropriate morpho-syntactic word forms during production.

### Functional organization within individual subjects

Compared to lesion studies, time-resolved cortical inhibition has the advantage of allowing for the function of closely neighboring sites to be profiled in detail within the same individuals. The nine sites that produced a selective deficit in the syntactic condition spanned a relatively small area measuring 6.6 ± 1.0 cm^2^ that was located within the posterior portion of P-STG below the temporoparietal junction (TPJ). This spatial colocalization was significantly unlikely to have been observed by chance given the number of tested sites (bootstrap test, *n*_perm_ = 1000, *p* < 0.01). Further, 12 of 14 participants received stimulation in sites outside of the P-STG (Supplementary Figure 2) but, of these, none of them displayed a selective deficit in functional morpheme production (i.e., a deficit on the syntactic but not lexical, semantic, or phonetic trials). The probability of observing this distinction in deficit between sites located within vs. outside the P-STG was highly unlikely to have been observed by chance (chi-square, *n* = 54, *χ*^2^ = 11.42, *p* = 0.0033).

**Fig. 3:**
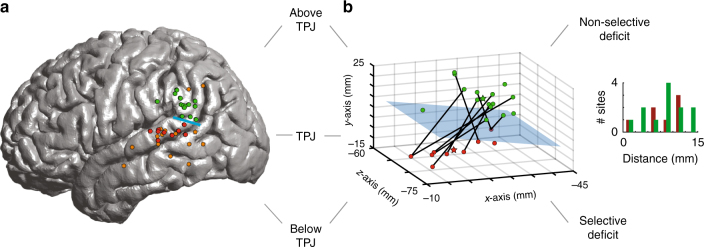
Partitioning of linguistic function within individual subjects. **a** Sites in which stimulation elicited a significant and selective deficit in the syntactic condition are displayed in red, whereas sites in which stimulation elicited a non-selective language deficit that involved more than one condition are displayed in green. Sites in which stimulation elicited a deficit but which did not reach the significance threshold (e.g., stimulation of the site elicited a deficit that was not reproducible on subsequent trials) are displayed in orange. **b** Stimulation sites are evaluated within individual subjects. Here, the anatomic locations of the stimulation sites and their relation to the TPJ plane are displayed in MNI space. The lines are used to indicate which sites were stimulated within the same subject. The bar graph in the inset represents the distance between the stimulation sites and the TPJ plane

As stimulation progressed above the TPJ within the same participants, we observed an abrupt loss of selectivity. Specifically, we identified 15 sites in 14 participants whose stimulation elicited a deficit during both the syntactic (binomial test, *n* ≥ 5, *p* < 0.0001, compared to the preoperative baseline) and control condition (binomial test, *n* ≥ 5, *p* < 0.0001, Fig. 3a). For these 15 sites, we observed similar results for the phonetic condition (binomial test, *n* ≥ 2, *p* < 0.0001), lexical condition (binomial test, *n* ≥ 2, *p* < 0.0001), and semantic condition (binomial test, *n* ≥ 2, *p* < 0.0001). Because all these sites displayed a deficit in the control condition, there were no differences in net percentage of errors between any condition pairs (chi-square, *χ*^2^>>0.05). In other words, stimulation of these sites produced a generalized, non-selective deficit. Sites above the TPJ in which stimulation led to a non-selective speech-production deficit colocalized to the supramarginal gyrus. They spanned an area measuring 8.1 ± 1.0 cm^2^ along the superior border of the TPJ (bootstrap test, *n*_perm_ = 1000, *p* < 0.01).

Spatially, sites in which stimulation produced a selective syntactic deficit vs. a non-selective deficit during production were closely apposed, lying within only 0.9 ± 0.1 cm of each other along the coronal axis. The midpoint between paired sites lied within ±0.2 cm of the TPJ (Fig. 3b). Moreover, sites in which stimulation produced a selective vs. non-selective deficit were significantly spatially distinct within individual subjects (Kolmogorov–Smirnov test of K-means centroid, *n* = 24, *p* = 0.003). In summary, within individual subjects, sites in which stimulation produced a selective deficit in the syntactic condition were consistently closely apposed to, yet significantly spatially distinct from, sites above the TPJ which were more broadly engaged in diverse linguistic computations.

### Neural encoding of functional morphemes across the TPJ

**Fig. 4:**
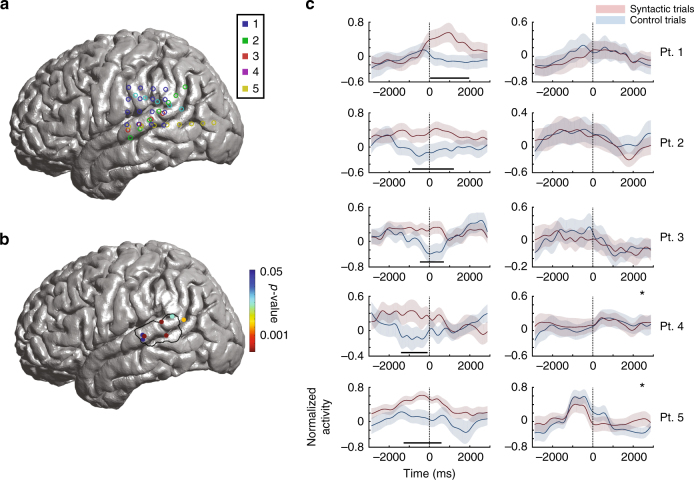
Neural responses during the production of functional morphemes. **a** Neural recording locations are color coded by participant. The recording grids were centered along the P-STG and were configured to match the available craniotomy openings. **b** Recording sites that demonstrated significant modulation during syntactic inflection compared to the control condition. The black outline here delineates the region identified by stimulation testing to produce a selective deficit on the syntactic condition. Only sites that displayed significant modulation are displayed. The degree of significance is displayed on the right. **c** Local field responses aligned to speech onset (time zero). Neural responses are displayed with their 95% confidence interval (±CI). Blue curves represent neuronal responses on control trials, whereas red curves represent neuronal responses on the syntactic condition trials. The horizontal lines represent time periods in which there was a significant divergence (“Methods”). The columns represent recording locations below (left) and above (right) the TPJ within the same individual subjects (sites close to the border are marked with an asterisk)

This apparent localization of function was also reflected in the neural recording data collected during the same paradigm. Given the above observations, intracranial recording electrode arrays were acutely implanted along the TPJ in five additional participants undergoing intraoperative neurophysiology (Fig. 4a; “Methods”). Of the 40 recording sites, seven sites across five participants (one or more sites per participant) demonstrated a difference in evoked local field potential (LFP) response between the syntactic and control conditions within 1.5 s of production of the target word (permutation test, *n*_perm_ = 3000, *p* < 0.05, Bonferroni corrected for two time points pre- and post production and for multiple sites; Fig. 4b, c). All seven sites lied within the P-STG and below the TPJ, and closely overlapped with the area in which functional morpheme-production deficit was found during stimulation. This spatial overlap was unlikely to have been observed by chance, given the tested stimulation and recording locations (Kolmogorov–Smirnov test, *n* = 94, *p* > 0.95; Fig. 4b).

Many of the sites that demonstrated a differential neural response to the syntactic vs. control conditions were also sensitive to the morphological properties of the target word. Specifically, five of the seven sites responded differentially depending on whether the target verb was in the present vs. past tense (71% sites; permutation test, *n*_perm_ = 3000, *p* < 0.05). Six of the seven sites responded differentially depending on whether the target noun was singular vs. plural (86% sites; permutation test, *n*_perm_ = 3000, *p* < 0.05). By comparison, none of the sites differentiated between “lower-level” aspects of word production such as adding vs. subtracting the suffix (0% sites, permutation test, *n*_perm_ = 3000, *p* < 0.05), and none of the sites differentiated between the phonetic and control conditions (0% sites, permutation test, *n*_perm_ = 3000, *p* < 0.05). These sites therefore appeared to encode morpho-syntactic, but not lower-level, properties of the to-be-produced words.

Lastly, to test whether the observed difference in neural response could have simply reflected the detection of incompatibility between the context (the preamble) and the target word, we additionally included a comprehension-only control manipulation, where the participants read sentences in which the final word was either semantically compatible or not compatible with the context (e.g., “We lit the candle” vs. “We lit the potato”; this task was not included in stimulation testing). No sites in the P-STG differentiated between these conditions (permutation test, *n*_perm_ = 3000, *p* < 0.05), ruling out general sensitivity to the detection of context incompatibility. In contrast, 4 of the 23 of sites above the TPJ (17%) showed reliable differentiation (permutation test, *n*_perm_ = 3000, *p* < 0.05). These neurophysiological observations during word production therefore appear to largely support our behavioral observations from stimulation.

### Spatiotemporal properties of neural encoding

**Fig. 5:**
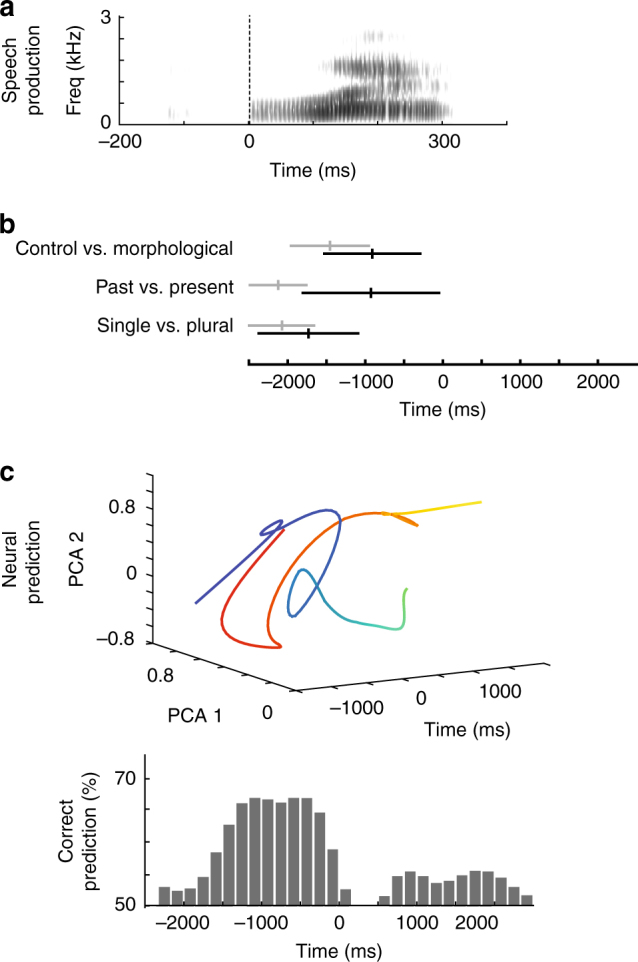
Predictive encoding of planned inflections prior to speech onset. **a** Example of a target word speech spectrogram. Neural data were aligned to speech onset (i.e., production of the target word) at time zero. **b** Time-course of neural modulation for the morpho-syntactic manipulations. The earliest divergence in activity across sites is displayed in gray, whereas peak divergence is displayed in black. The mean time points are marked by the vertical lines, whereas their standard deviations (across sites) are signified by horizontal lines. Note that although peak modulation based on syntactic inflection occurred shortly prior to speech onset, the earliest modulation based on the specific manipulation (e.g., past–present or single–plural) occurred up to 2 s prior to production. **c** Trial-by-trial decoding predictions over time for a representative subject. Below is the correct prediction performance for the syntactic vs. control trial conditions across all trials. Above are the representative principal components, again comparing the syntactic (red) and control (blue) trials

When did information about the planned inflection first arise in relation to the articulation of the target word? Temporally, the earliest significant divergence in evoked response between the syntactic and control conditions occurred 1.5 ± 0.5 s before word production, with peak modulation occurring at 0.9 ± 0.6 s before speech onset (Fig. 5a, b and Supplementary Figure 6). All seven sites significantly distinguished between the syntactic and control condition with average accuracies that ranged between 57.4 and 65.5% (Fisher discriminant, *H*_0_ = 50% syntactic vs. control condition, *n* ≥ 40, *p* < 0.05; Fig. 5c); and all sites, except one, were maximally distinguishing before speech onset. Peak differential response for plurality preceded that of tense by ~0.8 s (Fig. 5b).

**Fig. 6:**
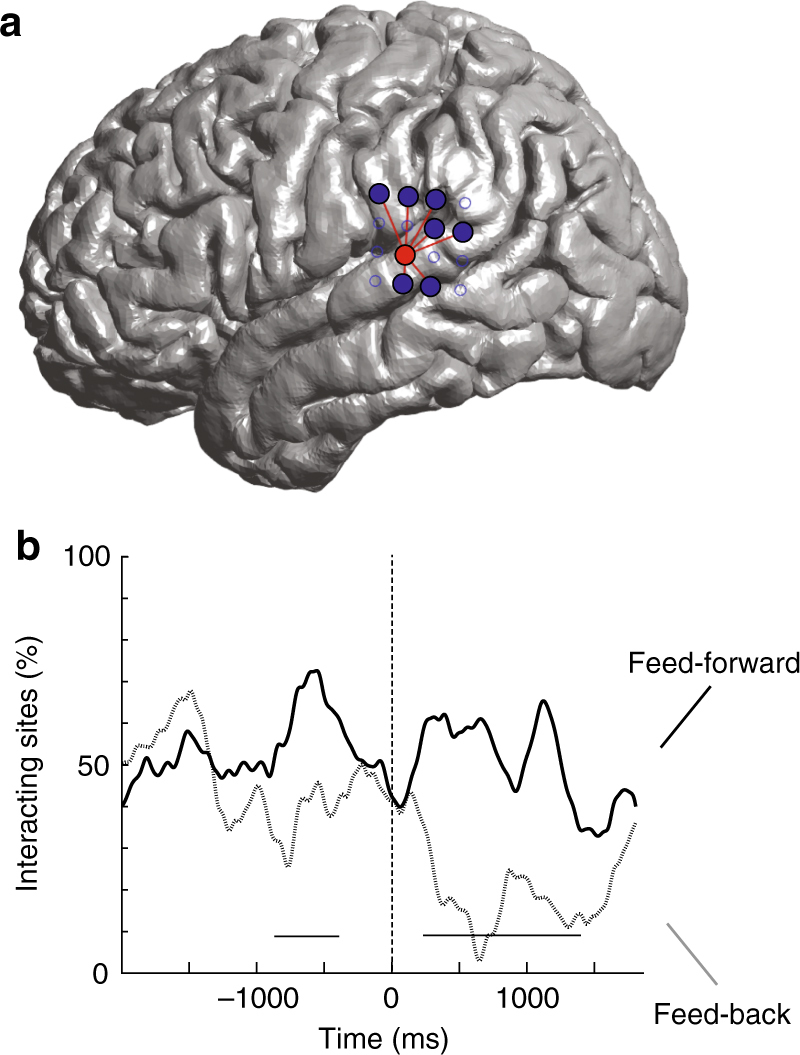
Interaction dynamics during the production of functional morphemes across the TPJ. **a** The spatial distribution of functional interactions for one representative recording site prior to articulation onset. **b** Percentage of sites that displayed significant functional interaction by time series analysis. Feed-forward interactions (i.e., from sites that differentiated between the syntactic and control conditions) are displayed in black and feed-back interactions are displayed in gray. The underlined areas represent time periods during which there was a significant difference in directional driving

We also examined the functional interaction between sites across the TPJ (Fig. 6a). The seven sites that distinguished between the syntactic and control conditions displayed a significantly stronger feed-forward compared to backward interactions with the sites above or near the TPJ (time series analysis; *n*_pair_ ≥ 6, *p* < 0.05). This divergence in forward vs. backward driving was first observed 0.8 ± 0.3 s prior to speech onset (Fig. 6b and Supplementary Figure 7), and ~0.7 s after the initial divergence in evoked response. Therefore, when taken together, both the neural encoding of planned inflections in the P-STG and the functional influence of the P-STG on sites above the TPJ appeared to shortly precede word production.

### Focal gray matter dysfunction disrupts morpheme production

Could our stimulation and neural recording results be explained by the connectivity of the P-STG to other downstream cortical sites such as the inferior frontal cortex^23-25^? In other words, would a focal gray matter lesion in the P-STG that largely spared the underlying white matter tracts be sufficient to produce a selective deficit in the production of functional morphemes?

**Fig. 7:**
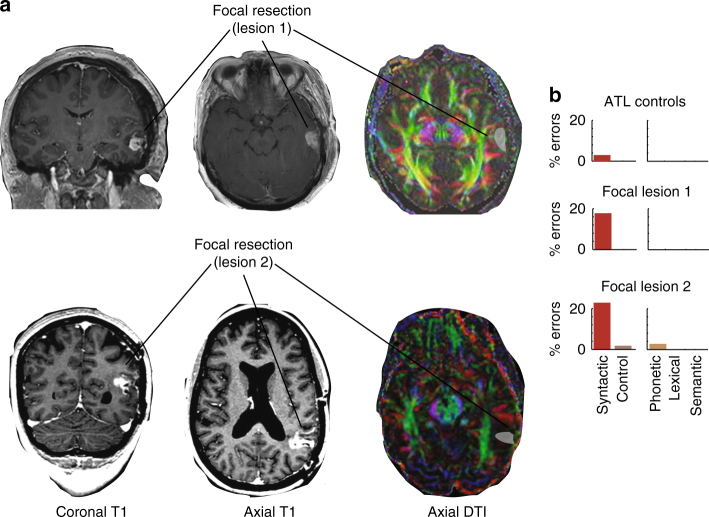
A focal P-STG gray matter lesion produces a selective deficit in functional morpheme production. **a** Coronal (left) and axial (middle) T1-weighted images show the resection site (in white) and the corresponding cortical ribbon of two patients with focal gray matter lesions. Diffusion tensor imaging (DTI, right) displays the underlying white matter tracts. **b** Performance of the two participants with a focal P-STG lesion (below) compared to six control participants who underwent extended anterior temporal lobectomies (ATL, above) in the dominant hemisphere

**Fig. 8:**
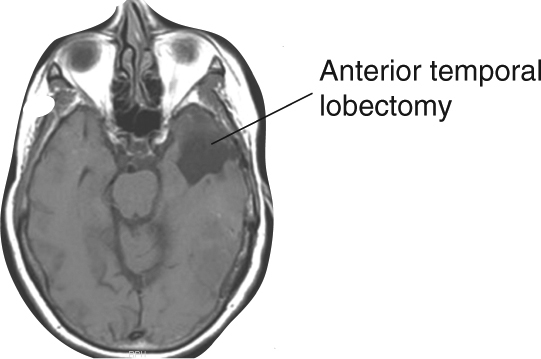
Anterior left temporal lobe resection. A representative participant who had undergone extensive resection of the left anterior inferior, middle and superior temporal gyri, and which did not involve the P-STG

To address this question, we retrospectively searched for individuals who had previously undergone resection of lesions that focally involved the P-STG, and identified two subjects (“Methods”) with a post-surgical resection area localized to the gray matter ribbon. The surgical resection cavity of these individuals measured 2.1 cm and 2.0 cm in maximal dimension, respectively, and was nestled between the inferior P-STG and superior temporal sulcus (Fig. 7a, b, T1 weighted). Moreover, less than 10% and 21% of the resection cavity contacted the underlying white matter, respectively, and we confirmed that there was no explicit involvement of transcortical tracts by diffusion tensor imaging (Fig. 7a, b, DTI). That being said, it should also be acknowledged that we cannot completely exclude the possibility that some of the fibers connecting the P-STG and the IFG had not been affected, as pre-lesional DTI data in the selected patients were not available. This is important given that parts of the IFG have long been argued to play a role in syntactic and morpho-syntactic processing^32,44,45,46–48^. Both patients had undergone surgery at least 6 months prior to participation in the study. To further control for general cognitive deficits post resection, we compared both participants’ performance with that of six other patients who had previously undergone extensive anterior temporal lobe (ATL) resections, which did not involve this part of the P-STG (Fig. 8).

Similar to the ATL patients, one patient with the P-STG lesion (patient 1) made no errors in the control, phonetic, lexical, or semantic conditions (0%, Fig. 7b). The other patient (patient 2) made errors on 2.5% and 10% of trials in the control and phonetic conditions, respectively, and none in the lexical and semantic conditions (Fig. 7b). In contrast to the ATL patients, both patients made significantly more errors in the syntactic condition (patient 1: 18% vs. 3%; patient 2: 28% vs. 3%). By comparison, no difference was observed when comparing regular and irregular forms (chi-square, *n* = 110, *χ*^2^ = 0.11, 1.41, *p* = 0.75, 0.24), or target words in which the suffix had to be added vs. subtracted (chi-square, *n* = 110, *χ*^2^ = 0.12, 0.14, *p* = 0.72, 0.71). With regard to noun and verb target words, patient 1 exhibited no difference (chi-square, *n* = 110, *χ*^2^ = 0.11, *p* = 0.75), whereas patient 2 performed worse on nouns than verbs (chi-square, *n* = 110, *χ*^2^ = 7.67, *p* = 0.006). This latter finding may be related to the more posterior location of this patient’s lesion, and concordant encroachment onto the inferior TPJ. Notwithstanding, a small gray matter lesion in P-STG below the TPJ appears to be sufficient to produce a morpho-syntactic deficit that is selective in nature and generalizes across different word manipulations.

## Discussion

Functional morphemes serve to convey precise word meanings and relationships among words, and thus provide a unique opportunity to study some of the most elementary building blocks and processes through which humans express complex concepts and ideas. Historically, language research has predominantly focused on language comprehension, in part due to the challenges associated with probing production. Because the same store of language knowledge is plausibly accessed both when we understand and produce words, studying language comprehension can inform our understanding of word and sentence production. Nevertheless, computations associated with the generation of linguistic utterances solve a fundamentally different problem from that of language comprehension: namely, to convey a specific idea. To do so, we have to utter a precise sequence of words where each word takes a particular morpho-syntactic form, and the words appear in a particular order (cf. the probabilistic and noisy representations that mediate language comprehension^12–14^). Recent application of extra- and intraoperative recording and stimulation approaches has begun to shed critical light on the articulation component of language production^6,27,28,49^. Here, we instead focused on an earlier component: morpho-syntactic encoding.

Using intraoperative stimulation, we identified a focal cortical area below the left TPJ within individual subjects whose transient inhibition selectively disrupted morphological word-form selection. This effect generalized across verbs (tense feature selection) and nouns (number feature selection), as well as across regular and irregular words. Critically, however, inhibition in these same sites did not affect other aspects of language production, like semantic and lexical retrieval, and articulation; nor did it affect basic language comprehension. Acute recordings in this area mirrored the inhibition results: many sites differentiated between the syntactic and control conditions, and additionally encoded the morphological form of the target word (e.g., the tense of the verb, or the plurality of the noun), but not lower-level properties of the word or task (e.g., suffix addition vs. subtraction). Importantly, we ruled out the possibility that some of these recording results may be due to simply detecting an incompatibility between the sentence preamble and the target word by showing that these sites do not differentiate between sentences that end in a predictable word vs. in a lexico-semantic violation.

Just across the TPJ border, superiorly and posteriorly to the region exhibiting selectivity for the production of functional morphemes, we observed sites with a strikingly distinct functional profile: their stimulation led to deficits across conditions. Within individuals, the morpho-syntactically-selective sites and sites whose stimulation elicited a broad linguistic deficit were notably closely apposed, lying within less than a centimeter of each other. Further, we found strong feed-forward interactions between the morpho-syntactically-selective sites and sites above the TPJ less than a second before speech onset.

Collectively, our observations suggest that sites lying within the P-STG immediately below the TPJ may implement some aspects of morpho-syntactic encoding during utterance planning, whereas upstream areas above the TPJ, along with the sites in the left inferior frontal lobe (IFL)^26–29^, likely support later stages of language production, including the assembly of the phonological representations and their articulation (e.g., in line with some existing proposals, like Roelofs^50^). In support of this, we find that two individuals with lesions confined to the cortical gray matter in this area, which largely spared underlying white matter tracts. Both patients displayed a selective functional morpheme-production deficit, which suggests that damage to P-STG is likely sufficient to cause lasting difficulties with functional morpheme production.

While damage to P-STG appears to be sufficient to produce a selective functional morpheme-production deficit, it is also likely that the dorsal tracts and/or the frontal cortical areas play a role in syntactic processing. For example, there are extensive anatomical connections between P-STG and frontal cortical language sites and prior studies have linked syntactic processing to both IFL and posterior temporal cortical areas^41,44,45,46–48^ as well as the dorsal stream tracts connecting them^51^. The current study, however, focused on P-STG and did not evaluate the role of the frontal lobe in the production of functional morphemes (Supplementary Figure 3). Consequently, the division of linguistic labor between P-STG and the inferior frontal areas that have been implicated in syntactic processing in both comprehension^32,46–48^ and production^44,45^ remain to be discovered. Similarly, future work may extend these findings to other production paradigms, including more naturalistic tasks, to test whether the focal nature of the results would generalize to more naturalistic contexts. Finally, our study did not include comprehension tasks that would require reliance on morpho-syntactic cues to derive the correct interpretation.

In summary, our results suggest that—at least within the cortical areas tested here—functional morpheme processing constitutes a discrete step in the series of computations necessary for word and sentence production. This encoding appears to be implemented focally within the posterior-most portion of the STG, below the TPJ, and these neural circuits exhibit strong selectivity for functional morpheme-production relative to other aspects of language production. Intriguingly, these results stand in contrast to what we generally know about morpho-syntactic processing during comprehension, where lexico-semantic and combinatorial (syntactic and semantic) processes appear to engage the same regions within the fronto-temporal network^18–20,52^, including when probed with temporally sensitive methods^53^. This difference is in line with distinct computational demands that comprehension and production impose, whereby production is inherently more precise and serialized than comprehension. The use of temporally resolved stimulation and direct intraoperative recordings allows to selectively tap different components of language within individual subjects and could therefore provide a more detailed circuit-based understanding of the functional architecture of language in the brain.

## Methods

### Participants

A total of 27 participants were evaluated (*n* = 14 for stimulation, *n* = 5 for recording, and *n* = 8 for lesion evaluation; Supplementary Table 1). The average lesion size was 69.6 ± 14.7 cm (s.e.m.). The most common pathology was GBM (*n* = 8), followed by astrocytoma (*n* = 4). All participants were native speakers with intact fluency, per preoperative testing (see below, paragraph 3).

### Participant selection and demographics

All study procedures were performed under approval and strict guidance by the Massachusetts General Hospital Internal Review Board (IRB). As detailed in paragraph 10 (“Intraoperative language task administration and data coding”), the study procedures were carefully designed so as not to perturb any aspect of the participant’s planned clinical care and to naturally integrate with their standardized neurophysiological testing. Participants involved in cortical stimulation and neural recordings were recruited from the same patient pool normally scheduled to undergo standardized intraoperative language mapping. The stimulation and recording studies were done in different sets of participants in order to minimize the duration of intraoperative testing. Patients considered for lesion evaluation (anterior or posterior temporal) were selected from a list of neurosurgical patients who had previously undergone craniotomy for lesion resection over a 2-year period. The specific indications for surgery and patient demographics are provided in Supplementary Table 1. An audio-visual system (microphone/speaker and video camera; BlackRock) was used to record all task events and align them to the neurophysiological data at millisecond resolution.

Prior studies have shown that subjects with underlying neural pathology but intact cognitive/language function can serve as an appropriate model for understanding normal neurophysiological processes^6,47,54–56^. Patients were screened for language function preoperatively in two steps. Toward this end, we first administered a standard preoperative language battery that is used for all neurosurgical patients at our institution irrespective of their participation in research (see ref.^46^ for additional details). Second, all prospective patients that agreed to participate in the study performed the target task preoperatively using a separate set of sentences and words from those employed intraoperatively. This included the control, syntactic, phonetic, lexical, and semantic conditions (with 40 trials per condition). Any patient who performed below 90% correct in any of these conditions was excluded. This preoperative testing was performed for all patients that underwent either (1) stimulation testing or (2) recordings. As detailed in the main text, participants exhibited a near-ceiling performance preoperatively across task conditions (>97.5% average correct).

Overall, we observed little difference between the participants based on demographics/pathology. Specifically, in the stimulation subset, we find no relationship between the presence of an elicited syntactic deficit and the following characteristics: lesion location (frontal vs. temporal vs. parietal; chi-square, *n* = 14, *p* > 0.1), size (larger vs. smaller than 100 cm^2^, chi-square, *n* = 14, *p* > 0.1; or size coded continuously, two-sample *t*-test, *t* = 0.85, *n* = 14, *p* = 0.41), pathology (benign vs. malignant; chi-square, *n* = 14, *p* > 0.1), and stimulation strength (two-sample *t*-test, *t* = −2.98, *n* = 14, *p* = 0.11). Similarly, in the recording subset, we found no relationship between the presence of neural modulation in the syntactic condition and lesion location, size, or pathology (chi-square, *n* = 14, *p* > 0.1). Please also see refs.^46–48,57^ for further discussion. Of note, patients with lesions directly involving the P-STG were automatically excluded from this study as patients with such lesions commonly have significant aphasia.

### Cortical stimulation mapping

The delivery of brief bipolar stimulation currents allows one to evaluate the time-causal relation between a focal area and function. As shown previously, it is safe, reproducible, and reversible^46–48,57^. Here, bipolar stimulation (4–10 mA; 900X143; Viasys Healthcare) was applied to the cortical surface using a two-prong probe with 10 mm spacing, leading to the transient disruption of cortical activity within an ~1 cm^3^ area. This procedure was performed in 14 patients undergoing tumor resection. Stimulations were given in 2 s runs with a 1 ms pulse width, at a frequency of 60 Hz. Stimulation commenced at the presentation of the target word and prior to speech production.

Stimulation within the available craniotomy proceeded in a grid-like arrangement, as allowed by the surgical constraints, with stimulation sites spaced ~1–2 cm apart. As stimulation was delivered, all stimulated sites were registered to the BrainLab stereotactic navigational system based on preoperative high-resolution MRI normalized to the Montreal Neurological Institute (MNI) 152 standard template. Due to variations in the size and location of craniotomy, the number of stimulated sites varied among the 14 participants (range: 3–14, mean: 8.07 ± 3.25 (SD)). All stimulation sites (*n* = 113) were first tested with ~1–2 syntactic trials to screen for regions eliciting any incorrect response (i.e., a mismatch between the target words and verbal response). To further allow for an unbiased evaluation of the stimulated sites per condition, we progressively increased the amplitude of stimulation from 4 to 10 mA until stimulation produced at least two consecutive target word errors. Thereafter, stimulation settings were then kept fixed for all trials and conditions. All stimulations were evaluated for after-discharge events, and any stimulation runs that elicited an after-discharge were excluded from analysis (these were rare, on the order of 5% or fewer across patients).

### Intraoperative neural recordings and data processing

Electrographic recordings of surface local field potentials (LFP) were performed using a Natus XLTEK neurophysiology work station on five participants undergoing tumor resection (see Supplementary Table 1 for more information). The signals were processed and amplified using a Nicolet v44 vEEG amplifier (Natus Neuroworks 8.4.1, Build 3538). Electrode arrays consisted of 1 cm spaced platinum iridium contacts and placed on the cortical surface of the left hemisphere and confirmed to have stable impedances. All electrodes were referenced to a common ground (low-impedance ground wire in the subcutaneous galia and outside the skull). Colocalization of the electrodes with preoperative high-resolution MRI was performed using the BrainLab navigational system. As in the stimulation study, due to variations in the size and location of craniotomy, the number and location of electrodes varied among the five participants, with one patient having a 4×4 grid, and the remaining four having an 8×1 strip.

Recorded LFP data were collected at 200 Hz for three patients. Because of an upgrade to the Natus system during our study, two patients had recordings at 256 Hz. This update did not affect our ERP analyses as they did not involve the time–frequency domain. Off-line signal processing was performed with custom-written scripts in MATLAB version 8.5.0 (Mathworks, Inc; see further detail below^58–60^).

The LFP traces were notch-filtered at 60 Hz to remove the land-line artifact. Occasionally (depending on the operating room), we encountered noise at 20 or 105 Hz, which was likely due to instruments in the operative field. These were similarly notch-filtered in two of the patients at the appropriate frequencies. These filters did not affect our results, which represented changes in the ERP rather than changes in the time–frequency domain. To optimize the signal-to-noise ratio, we normalized the time series to the baseline (i.e., time periods between sentences) by subtraction on a per-channel basis. An example of this workflow is provided in Supplementary Figure 5.

### Intraoperative language task administration and data coding

For the stimulation component, each trial was coded by three testers. In the operating room, tester #1 recorded the location and time of each stimulation, tester #2 recorded the preamble and the target word presented to the patient, and tester #3 recorded the participant’s verbal output. Tester #3 was blind to the condition since they did not know which target word was presented. For the recording component, the workflow was the same, with the exception of tester #1, who now marked the location and positioning of the ECoG array.

With regard to the errors themselves, the participant’s responses were coded as either correct or incorrect (1 or 0). Here, responses were coded as incorrect if there was a mismatch between the correct answer and the verbal response of the participant. For example, the participant’s response would be coded as incorrect if the target word was “talk”, the correct answer was “talked” but the participant responded by saying “talk”. Alternatively, the participant’s response would be also coded as incorrect if the target word was “talked”, the correct answer was “talked” but the participant responded by saying “talk”. While the answer is incorrect in both cases, an error in both would indicate that stimulation in this area was not associated with a selective morpho-syntactic deficit. In other words, we took mismatch between the correct target word and the uttered word as an incorrect response—e.g., whether the mismatch was because of a missing suffix, inappropriately added suffix or incorrectly articulated word. Therefore, the type and selectivity of the deficit was defined by the condition in which the incorrected responses were found.

### P-STG lesion patient identification and analysis

We searched electronic patient medical records at Massachusetts General Hospital of >300 craniotomies with post-surgical resection areas in the temporal region in a 2-year period (2013–2015). We identified patients that had apparent focal gray matter lesions localized to the P-STG. We then examined each patient’s preoperative MRI, and obtained an apparent diffusion coefficient at each voxel and reconstructed the diffusion tensor by multi-linear regression across multiple images. The space path was constructed using a standard Frenet–Serret approach. Fibers were color coded based on their anisotropy and plotted in relation to the lesion location. Any fibers that were in contact with the lesion (i.e., fiber/voxel overlap) were considered to involve the lesion. Of these, two patients were found to have focal lesions limited to the gray matter. They were then post-operatively tested for language function using the language task used in the stimulation and recording studies. In addition, six patients with anterior temporal lobectomies were selected as controls and similarly tested post operatively.

### Cortical stimulation analysis

We used a standard binomial test to identify cortical sites in which stimulation elicited errors at a rate higher than expected by chance, within individuals, based on their preoperative testing. Resulting significance values were Bonferroni corrected for multiple sites (*p* < 0.0001). A Chi-square analysis was used to assess differences in performance between the levels of each condition (e.g., noun vs. verb) for all tested sites across individuals (*p* < 0.01).

Spatial locations were evaluated after conversion to the MNI standardized space. To evaluate the spatial distribution of sites that produced a particular deficit, we used K-means analysis to calculate their area and centroid. The standard error of the fit was estimated by comparing the Cartesian distance of the individual points to the centroid. Significant differences in spatial location between sites were assessed via a two-dimensional Kolmogorov–Smirnov test (*p* < 0.05). The probability of sites being colocalized within a particular spatial distribution was assessed by bootstrap analysis (*p* < 0.01). This was achieved by scrambling the stimulated sites (*n*_perm_ = 1000; Fig. 1c), such that a distribution of coordinates, and in turn, a K-means centroid, was attained for each site. A Kolmogorov–Smirnov test was then conducted between the bootstrapped K-means centroids and sites in which stimulation produced a deficit. Significance was set at *p* < 0.05.

### Neural recordings analysis

The LFP signals were time locked to speech onset (*t* = 0s) using a microphone that was synchronized to the Natus XLTEK neurophysiology work station at millisecond resolution (1 kHz). The data were then evaluated, for each spoken word, over the timespan ranging from −2.5 s (prior to speech onset) to 2.5 s (post speech onset).

Presence of significant differential response across conditions was determined by bootstrap analysis for each channel separately, as described previously^61,62^. Here, a permutation analysis was performed by randomly shifting the *n* time stamps of vocalized response across 3000 permutations (±1–50 s), such that a distribution of time stamps was attained. The difference between the respective averages across conditions (e.g., syntactic vs. control) was then compared to the distribution of differences obtained from the permutation (*n* = 3000). Significance for differential activity was set at a *p* < 1 × 10^–5^, Bonferroni corrected for multiple comparisons. Therefore, similar to prior reports, this procedure ensured that the change in evoked response was due both to (1) differences between conditions, and (2) word onset.

A Fisher discriminant was used to determine when reliable variations in LFP occurred and whether they could discriminate individual trials with respect to condition (e.g., syntactic vs. control) for each channel separately^63,64^. Here, we defined the predicted condition “*y*” as,

$$y = \mathop {{\arg }}\limits_{y = 1, \ldots ,\;K} {\mathrm{min}}\mathop {\sum }\limits_{k = 1}^K P\left( {k|x} \right)M\left( {y{\mathrm{|}}k} \right)$$

where *K* is the number of conditions, *P*(*k*|*x*) is the posterior probability of condition *k* for observation *x* and *M*(*y*|*k*) is the cost of misclassifying *x* as *y* when the actual condition is *k*. A Fisher model was completed for averages of normalized activity taken across successive 0.5 s windows. To ensure independence of training data, models were trained on 80% of the data and tested on the remaining 20% of the data.

Finally, in the time series analysis, interactions between sites were defined based on whether changes in neural activity of one site were predictive of upcoming changes in activity of another site. Optimal time lags were determined by Bayesian information criterion, with maximum lag length set at 0.2 s. Significance was set at *p* < 0.05. Causality was determined for a 200 ms time series window of the difference between average opposing conditions (e.g., syntactic – control conditions) for every possible pair of recorded sites. Both forward and reverse driving interactions were considered (e.g., Channel A → Channel B, and Channel B → Channel A). This analysis was performed for these windows shifted in 2 ms increments on a per-channel basis, such that a time series of percent significant interactions was calculated for each channel. For the analysis itself, we consider a bivariate linear autoregressive model of two variables *X*_1_ and *X*_2_:

$$X_1\left( t \right) = \mathop {\sum }\limits_{j = 1}^p A_{11,j}X_1\left( {t - j} \right) + \mathop {\sum }\limits_{j = 1}^p A_{12,j}X_2\left( {t - j} \right) + E_1(t)$$

$$X_2\left( t \right) = \mathop {\sum }\limits_{j = 1}^p A_{21,j}X_1\left( {t - j} \right) + \mathop {\sum }\limits_{j = 1}^p A_{22,j}X_2\left( {t - j} \right) + E_2(t)$$

Here, *p* is the maximum number of lagged observations included in the model (the model order), the matrix *A* contains the coefficients of the model (i.e., the contributions of each lagged observation to the predicted values of *X*_1_(*t*) and *X*_2_(*t*), and *E*_1_ and *E*_2_ are residuals (prediction errors) for each time series. Therefore, if the variance of *E*_1_, for example, is reduced by the inclusion of the *X*_2_, then it can be concluded that *X*_2_ influences *X*_1_. Appropriate model selection was made by Bayesian information criteria. Lastly, we confirmed that there was no relation between the magnitude of recorded signal and strength of interaction across the tested sites (Pearson’s correlation, *n*_conditions_ = 7, *r* = 0.163, *p* > 0.05). Here, a Pearson’s value was calculated between the Δ activity between the relevant conditions (e.g., syntactic vs. control) and the Δ% of channels predicted by forward vs. reverse driving.

### Data availability

The data and primary codes that support the findings of this study are available from the corresponding author on reasonable request.

## Electronic supplementary material

Supplementary Information
