# Supplementary material for: Neural encoding and production of functional morphemes in the posterior temporal lobe
Source: Nat Commun. 2018 May 14;9:1877. doi: 10.1038/s41467-018-04235-3 (PMC5951905; doi:10.1038/s41467-018-04235-3)
Supplement: Supplementary file 1 — Supplementary Information [file 41467_2018_4235_MOESM1_ESM.pdf]

Supplementary Information

Neural encoding and production of functional morphemes in the posterior temporal lobe

Daniel K. Lee[1*], Evelina Fedorenko[2,3,4*], Mirela V Simon[5], William T Curry[1], Brian Nahed[1], Dan P Cahill[1], Ziv M Williams[1,6,7]

1. Department of Neurosurgery, Harvard Medical School, Massachusetts General Hospital, Boston MA
2. Department of Psychiatry, Harvard Medical School, Massachusetts General Hospital, Boston MA
3. Harvard Program in Speech and Hearing Bioscience, Cambridge MA
4. Massachusetts Institute of Technology, McGovern Institute for Brain Research, Cambridge MA
5. Department of Neurology, Harvard Medical School, Massachusetts General Hospital, , Boston MA
6. Harvard-MIT Division of Health Sciences and Technology, Boston MA
7. Harvard Medical School, Program in Neuroscience, Boston MA

* These authors contributed equally to the work

SUPPLEMENTARY TABLES

Patient	Testing	Lesion Location	cm^2	Pathology
1	Stimulation	Anterior frontal	121	Oligoastrocytoma
2	Stimulation	Superior temporal	61	Oligoastrocytoma
3	Stimulation	Temporal, parietal	28	Amyloid Angiopathy
4	Stimulation	Frontal, temporal	293	Astrocytoma
5	Stimulation	Temporal	141	Glioblastoma
6	Stimulation	Temporal	138	Astrocytoma
7	Stimulation	Temporal	183	Oligodendroglioma
8	Stimulation	Temporal	83	Astrocytoma
9	Stimulation	Parietal	57	Astrocytoma
10	Stimulation	Temporal	27	Ganglioglioma
11	Stimulation	Temporal	184	Epidermoid Cyst
12	Stimulation	Temporal	50	Oligoastrocytoma
13	Stimulation	Temporal, parietal	14	Glioblastoma
14	Stimulation	Temporal	27	Metastatic Melanoma
15	Recording	Temporal	5	Metastatic Melanoma
16	Recording	Frontal, temporal	46	Glioblastoma
17	Recording	Temporal, parietal	30	Glioblastoma
18	Recording	Temporal, parietal	54	Glioblastoma
19	Recording	Temporal	19	Glioblastoma
20	Lesion	Temporal*	2	Glioblastoma
21	Lesion	Temporal*	2	Glioblastoma
22	Lesion	Temporal	3	Glioblastoma
23	Lesion	Temporal	9	Astrocytoma
24	Lesion	Temporal	17	Metastatic Carcinoma
25	Lesion	Temporal	n/a	Mesial temporal sclerosis
26	Lesion	Temporal	n/a	Mesial temporal sclerosis
27	Lesion	Temporal	n/a	Mesial temporal sclerosis

Supplementary Table 1. Testing modality, lesion location and size, and underlying pathology. Participants are color-coded by testing modality: stimulation (light green), recording (light blue), or post-operative lesion evaluation (*the first two patients had P-STG lesions). Lesion size is represented in cubic centimeters, and approximate anatomical locations are provided. All lesions involved the left dominant hemisphere. As noted in the Supplement text, there was no difference in the main neurophysiological findings based on patient demographics or pathology.

Condition	Target	Reg/Irreg	Number | Tense	Sentence preamble	Input word	Correct production
Control	Noun	Regular	Singular	*This is the …*	*apple*	***apple***
Syntactic	Noun	Regular	Plural	*These are the …*	*apple*	***apples***
Syntactic	Noun	Regular	Singular	*This is the …*	*apples*	***apple***
Control	Noun	Regular	Plural	*These are the …*	*apples*	***apples***
Control	Noun	Irregular	Singular	*This is the …*	*scarf*	***scarf***
Syntactic	Noun	Irregular	Plural	*These are the …*	*scarf*	***scarves***
Syntactic	Noun	Irregular	Singular	*This is the …*	*scarves*	***scarf***
Control	Noun	Irregular	Plural	*These are the …*	*scarves*	***scarves***
Control	Verb	Regular	Future	*Today we will …*	*talk*	***talk***
Syntactic	Verb	Regular	Past	*Yesterday, we …*	*talk*	***talked***
Syntactic	Verb	Regular	Future	*Today we will …*	*talked*	***talk***
Control	Verb	Regular	Past	*Yesterday, we …*	*talked*	***talked***
Control	Verb	Irregular	Future	*Today we will …*	*run*	***run***
Syntactic	Verb	Irregular	Past	*Yesterday, we …*	*run*	***ran***
Syntactic	Verb	Irregular	Future	*Today we will …*	*ran*	***run***
Control	Verb	Irregular	Past	*Yesterday, we …*	*ran*	***ran***

Supplementary Table 2. **Syntactic manipulations.** Examples of the different types of trials in the critical syntactic condition, where participants had to morpho-syntactically manipulate the target word prior to production (targets shown in red) and the control condition, where participants had to simply read the target word out loud (targets shown in green). The trials varied in whether the target word was a) a noun or a verb, b) morphologically regular or irregular, and c) singular or plural for nouns, or in the past vs. future tense for verbs. The latter manipulation resulted in some cases where a morpheme had to be *added* and other cases where it had to be *subtracted* to produce the correct word.

SUPPLEMENTARY FIGURES

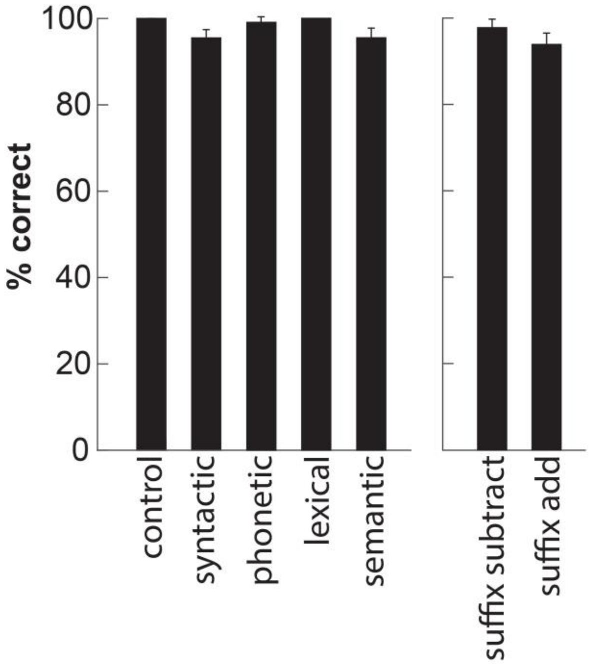

Supplementary Figure 1. Pre-operative baseline performance for participants in the stimulation and recording components. =Performance was near ceiling across conditions across patients (*n*=19 total). To test for potential effects of task demands on performance, we additionally compared pre-operative baseline performance to performance during stimulation based on (i) suffix addition vs. subtraction comparison, and (ii) evaluation of between-condition differences in pre-operative performance (i.e., 2-4% error vs. 0-2% error). As detailed in the main text, the results suggested that there was little variation in effect in the P-STG based on task demands. Error bars indicate +/- 95% CI s.e.m..

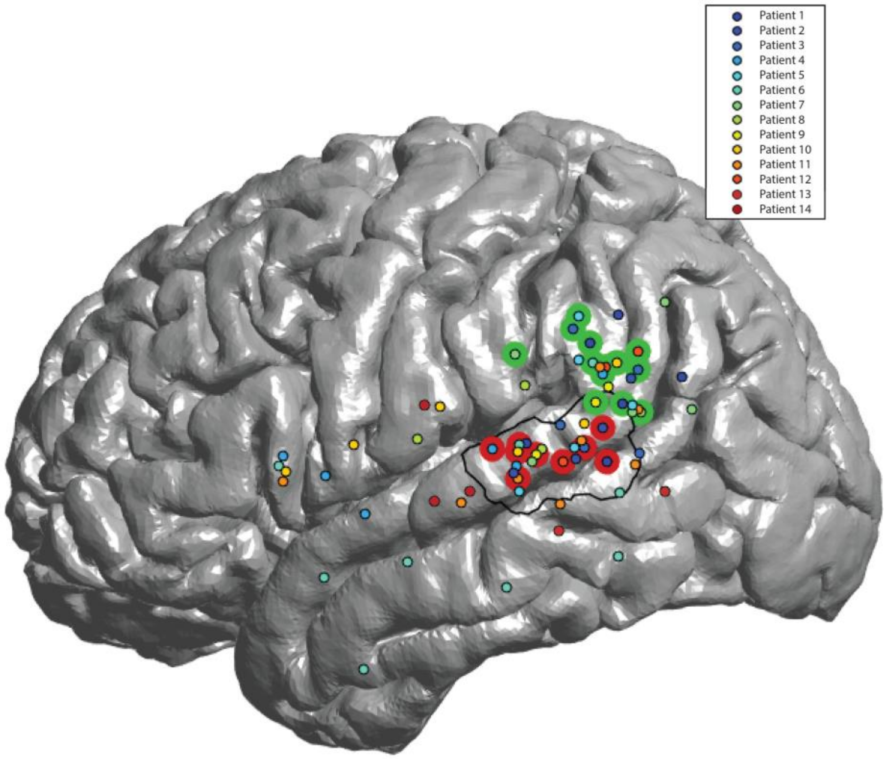

Supplementary Figure 2. Stimulated sites and elicited deficits on a patient-by-patient level. 54 sites were tested across 14 participants, transformed here to a standard MNI152 cortical atlas. Stimulation sites by participants are color-coded, as indicated in the legend. Sites eliciting a selective morpho-syntactic deficit in functional morpheme production vs. a global linguistic deficit are circled in red and green, respectively. Here, 9 of the 14 patients had specific access to the P-STG.

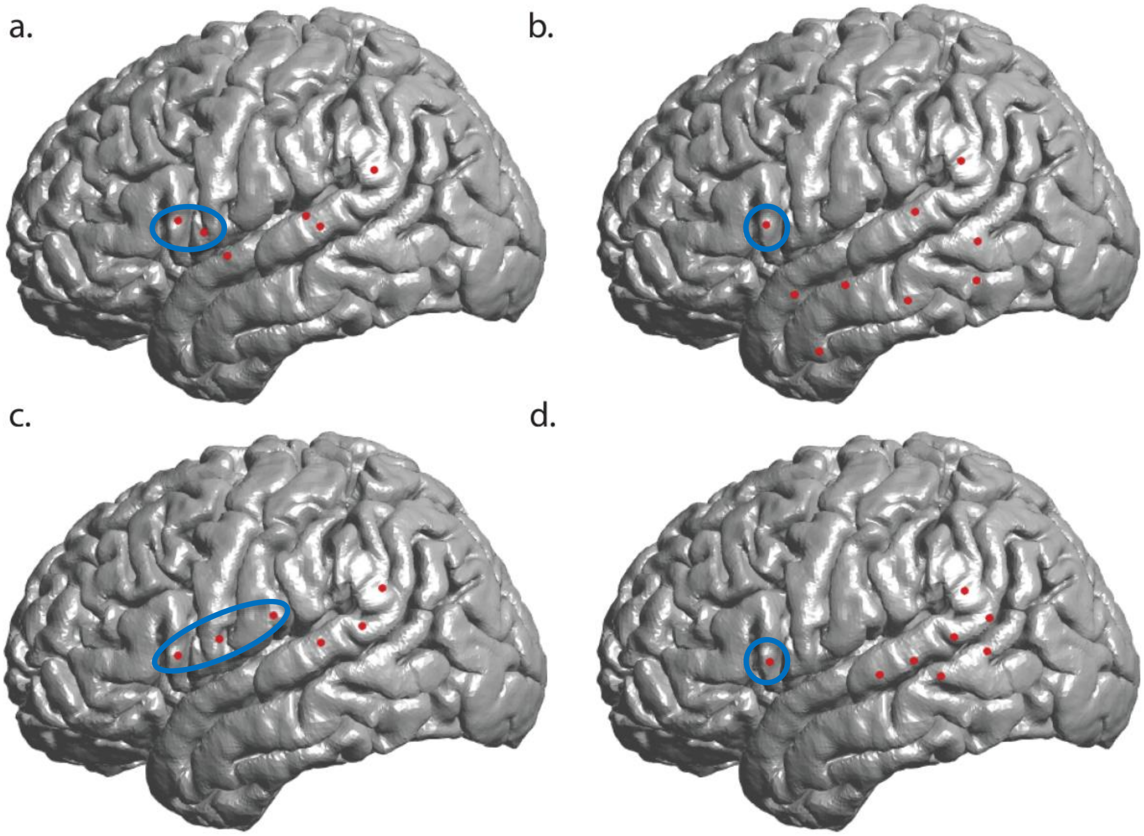

Supplementary Figure 3. Participants with anterior stimulation sites. Four of the 14 participants had craniotomies which covered a small portion of the inferior frontal gyrus. The sites tested in these patients are colored in red. Sites that involved frontal cortex are circled.

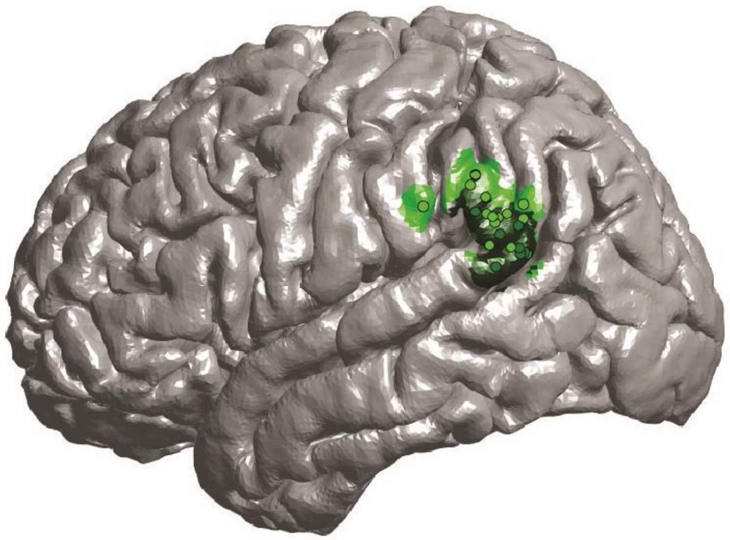

Supplementary Figure 4. Spatial distribution of deficits during lexical and semantic conditions. Stimulation sites that produced a deficit during the both the lexical and semantic conditions are displayed in light green. Sites that produced a deficit during the lexical but not semantic condition are shown in dark green. The shaded regions are calculated based on an average 1 cubic centimeter area estimated to be affected by focal stimulation.

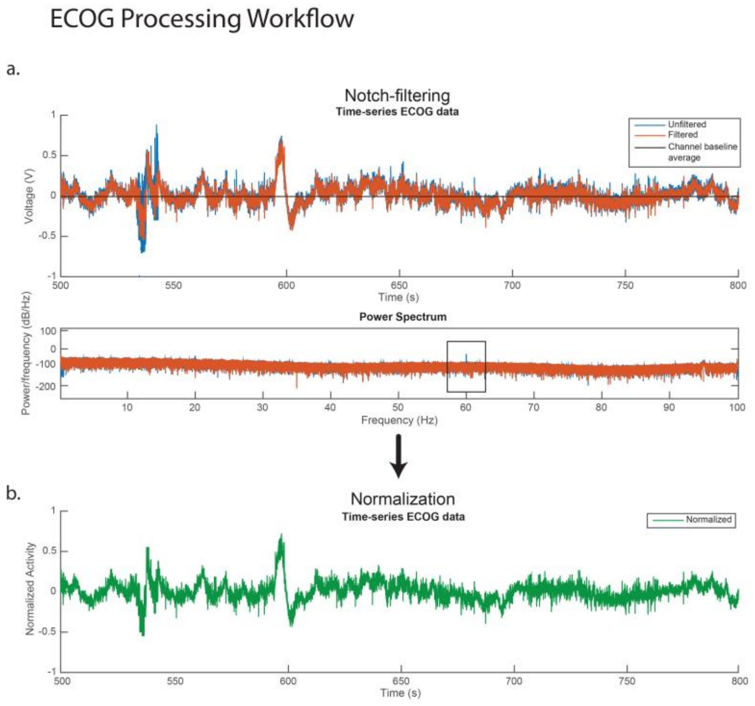

Supplementary Figure 5. ECOG processing workflow. Electrocorticography data were notch-filtered and normalized for each channel separately. The raw signals (blue) were notch-filtered at 60 Hz (orange) to remove land-line noise, and, in some patients, at 105 Hz and 20 Hz, due to instruments in the operative field. To maximize the signal-to-noise ratio, data were normalized by subtraction of the average baseline (i.e. time periods between trials) (green) for each channel.

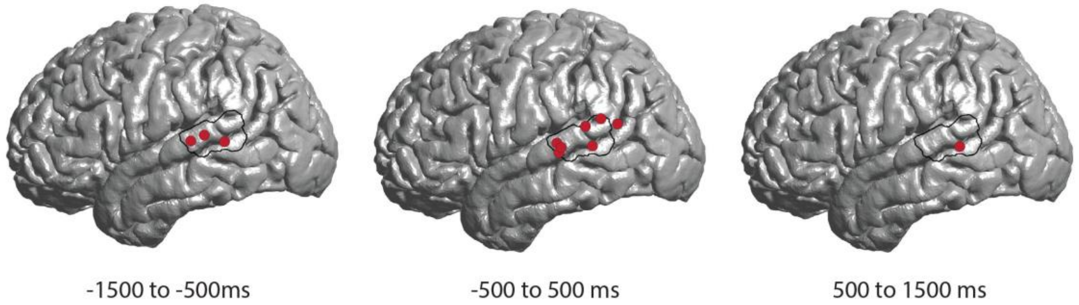

Supplementary Figure 6. The spatial-temporal activity dynamic of syntactic-sensitive sites. The times are based on a 1 second window centered before, during, and after speech onset (t=0). Any site with significant differentiation between syntactic and control conditions (permutation test, n_{perm}=3000, p<0.05) within the 1 second window are drawn.

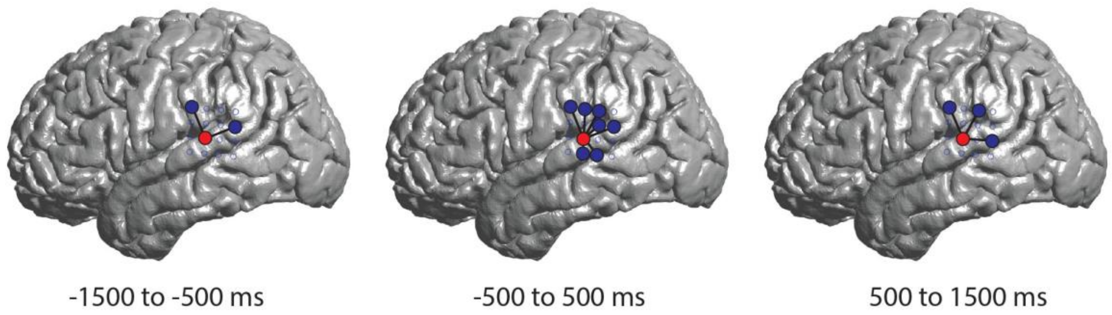

Supplementary Figure 7. Time-course and location of functional interactions. The results from a sample participant of the functional interactions observed between a site within the P-STG (red) and other neighboring sites (blue) over time. Only significant interactions are shown (time-series analysis, n_{pair}=15, p<0.05). The times are based on a 1 second window centered before, during, and after speech onset (t=0).
